# Dengue fever as a potential cause of sickle cell intrahepatic cholestasis: A report of two cases

**DOI:** 10.1590/0037-8682-0010-2021

**Published:** 2021-07-12

**Authors:** Leonardo Rodrigues de Oliveira, Ana Laura Castro Costa, Paula Veloso Almeida, Luzia Beatriz Ribeiro Zago, Vanessa Afonso da Silva, Sheila Soares-Silva

**Affiliations:** 1 Universidade Federal do Triângulo Mineiro, Serviço de Hematologia e Hemoterapia, Uberaba, MG, Brasil.

**Keywords:** Dengue, Sickle cell disease, Endothelial cells

## Abstract

Sickle cell intrahepatic cholestasis is a potentially fatal syndrome characterized by jaundice, painful hepatomegaly, and organ dysfunction. Two cases of sickle cell intrahepatic cholestasis associated with dengue fever were described. Endothelial damage/dysfunction is a mechanism involved in severe hepatobiliary complications related to sickle cell diseases. However, the reasons for the lack of increase in the admission of patients with sickle cell disease having severe acute hepatobiliary complications triggered by endothelial damage/dysfunction due to dengue fever remain unknown. This study describes the first association between sickle cell intrahepatic cholestasis and dengue fever.

## INTRODUCTION

Sickle cell disease (SCD)-associated hepatobiliary complications include acute and chronic manifestations of distinct physiopathology and severity. Among the acute hepatobiliary complications, sickle cell intrahepatic cholestasis (SCIC) is a potentially fatal syndrome characterized by jaundice, painful hepatomegaly, and organ dysfunction^1^ ^-^ ^3^. Predisposing conditions for a vaso-occlusive crisis such as dehydration, infection, and trauma may be potential causes of SCIC.

Two cases of SCIC associated with dengue fever have been reported. This is the first report that describes this association, after performing a thorough search in PubMed and Google Scholar databases for English language articles published from 2001 to 2020 using the terms “*Sickle cell anemia*”, “*Sickle cell disease*”, “*Cholestasis*”, “*Intrahepatic cholestasis*” and “*Dengue*”. 

## CASE REPORT

### Case 1

A 16-year-old female patient was admitted during the summer with a 4-day history of diffuse myalgia, retro-orbital headache, abdominal pain, fever, and chills. On admission, the patient looked pale and icteric, with tachycardia (123/min), palpable liver 1 cm below the right costal border, and the spleen was not palpable. The patient’s blood pressure was normal. Arterial oxygen saturation in room air was 99%. The patient had Sβ^+^-thalassemia (genotype S/thal β), and the average laboratory data from outpatient follow-up was serum hemoglobin concentration (Hb) 11.7 g/dL, mean corpuscular volume 62 fL, leucocyte count of 6.7 × 10^9^/L, and platelet count of 123 × 10^9^/L. The patient did not use hydroxyurea.

The initial laboratory test results are presented in Table 1. Metabolic acidosis was detected (pH 7.29; pCO2 28 mmHg; HCO3 13.8 mmol/L). Serology for viral hepatitis (A, B, and C) was non-reactive. Abdominal ultrasound revealed hepatosplenomegaly, moderate ascites, and bilateral pleural effusion without detection of gallstones or dilatation of the intrahepatic and extrahepatic bile ducts. Based on the local epidemiological, clinical, and laboratory data for a SCD patient along with serological confirmation of dengue (IgM reagent - risk group D according to the criteria of the Brazilian Ministry of Health)^4^, the diagnosis of SCIC associated with dengue (dengue hemorrhagic fever) was considered. The patient was treated with vigorous hydration, analgesia, transfusion of packed red blood cells to achieve a target hemoglobin S (HbS) concentration < 30% (exchange transfusion), and intravenous antibiotic therapy (ceftriaxone 2 g/day).

**TABLE 1:** Relevant laboratory data.

**Laboratorial Parameter *(normal range)***	Case 1		Case 2	
	Admission	Day 2	Admission	Day 3
Hemoglobin concentration (12-16 g/dL)	7.8	6.0	7.6	5.5
Leukocyte count (4-12 × 10^9^/L)	27.6 × 10^9^/L	-	12.2 × 10^9^/L	9.8 × 10^9^/L
Neutrophils (%)	83%	-	76%	79%
Lymphocytes (%)	6%	-	10%	14%
Platelet count (150-450 × 10^9^/L)	24 × 10^9^/L	-	714 × 10^9^/L	185 × 10^9^/L
Reticulocyte count (25-100 × 10^9^/L)	12 × 10^9^/L	-	93.7 × 10^9^/L	7.6 × 10^9^/L
Conjugated bilirubin (< 0.4 mg/dL)	2.4	2.1	1.0	3.4
Unconjugated bilirubin (< 0.8 mg/dL)	0.9	1.1	0.54	1.7
Aspartate aminotransferase (< 40 U/L)	10,423	-	26	2,625
Alanine aminotransferase (< 33 U/L)	5,895	-	21	1,807
Serum lactate dehydrogenase (135-225 U/L)	5,925	8,823	272	2,493
Serum creatinine (< 1 mg/dL)	1.34	2.2	0.67	-
Blood urea nitrogen (< 50 mg/dL)	42	75.7	40	-
Activated partial thromboplastin time (25-34 sec.)	40.5 s	44.9 s	-	77 s
Prothrombin activity (70-100%)	26.1%	21.7%	-	41%

Twelve hours after admission, the clinical condition worsened with the onset of respiratory distress, drowsiness, and intensification of abdominal pain with rapidly increasing hepatosplenomegaly (spleen and liver palpable at the level of the iliac crests). Laboratory parameters deteriorated with intensifying anemia, elevated LDH, and kidney and liver dysfunction (Table 1). Further management included maintenance of packed red blood cell transfusion (achieved HbS 19.8%) and supportive measures such as hemodialysis and mechanical ventilation in the intensive care unit. There was worsening organ failure, and the patient died eight days later.

### Case 2

A 24-year-old woman was admitted for a 1-day history of diffuse myalgia, arthralgia, retro-orbital headache, vomiting, and fever. The patient was pale, icteric, and tachycardic (108/min). The patient’s blood pressure was normal. The liver and spleen were not palpable. Arterial oxygen saturation in the room air was normal (98%). The patient had sickle cell anemia (genotype SS) with irregular outpatient follow-up and no history of hydroxyurea intake. The average laboratory data from outpatient follow-up showed: Hb 8 g/dL, leukocyte count of 20.5 × 10^9^/L, and platelet count of 612 × 10^9^/L.

Upon admission, laboratory tests revealed findings (Table 1). Suspected classic dengue fever was confirmed by detection of the nonstructural protein 1 (NS1) antigen test (risk group C), and the diagnosis of sickle cell crisis associated with dengue fever was considered. The initial treatment consisted of vigorous hydration, analgesia, and intravenous ceftriaxone (2 g/day).

Two days after admission, there was clinical deterioration characterized by drowsiness, painful hepatomegaly (palpable liver 6 cm below the right costal border), and respiratory failure. Follow-up laboratory tests detected a drop in serum hemoglobin, reticulocytopenia, a marked drop in platelet count, an increase in LDH, and acute renal dysfunction (Table 1). Tests for renal function were normal. Serology for viral hepatitis (A, B, and C) was negative. Abdominal ultrasonography revealed hepatomegaly, suprahepatic vein ectasia, single calculus (1.8 cm) in the gallbladder with no inflammatory signs, and no dilatation of intrahepatic and extrahepatic bile ducts. Due to the marked clinical and laboratory deterioration, a probable diagnosis of SCIC was considered, and support with packed red blood cells (exchange transfusion until HbS reached 28%) and non-invasive ventilation was instituted. Subsequently, there was a progressive clinical improvement, and the patient recovered completely.

## DISCUSSION

SCIC is an acute and severe complication with high mortality in patients with SCD, especially in patients with sickle cell anemia and Sβ-thalassemia^5^ ^-^ ^8^; however, the pathophysiology remains unclear^5^ ^,^ ^7^. The possible mechanism involves a sequence of events that culminate in liver dysfunction and includes adhesion of deformed erythrocytes to the vascular endothelium, obstruction and congestion of hepatic sinusoids, hypoxemia, ballooning, ischemia, and death of hepatocytes^1^ ^-^ ^3^ ^,^ ^5^ ^-^ ^7^.

The diagnosis of SCIC should be suspected in patients with SCD and non-obstructive cholestasis, usually accompanied by marked elevation of transaminases and painful hepatomegaly. SCIC is associated with acute organ dysfunction, especially but not exclusively restricted to renal and hepatic dysfunctions^6^ ^,^ ^9^. Serum concentrations of transaminases above 1,000 U/L are commonly observed^1^. Differential diagnoses such as obstructive cholelithiasis, obstructive choledocholithiasis, viral hepatitis, and autoimmune hepatitis should be excluded^2^ ^,^ ^6^ ^,^ ^7^. In severe cases, acute and multiple organ functional deterioration may arise associated with coagulopathy, altered mental status, and acute renal and liver failure^2^ ^,^ ^5^ ^,^ ^7^.

Treatment has not been precisely defined by prospective studies^2^ ^,^ ^6^. Therapeutic interventions for SCIC include hydration, analgesia, oxygen supplementation, antibiotic therapy (when indicated), and blood component transfusion (packed red blood cells, fresh frozen plasma). Exchange transfusion to reduce the proportion of HbS decreases the sickling process, and is the predominant therapeutic intervention despite the absence of studies defining optimal values for HbS^6^ ^-^ ^8^. Fresh frozen plasma transfusion can be used to correct coagulopathy^3^.

Data from the Brazilian Ministry of Health published in 2018 reveals that there are between 60,000-100,000 SCD patients in Brazil^10^. An epidemiological bulletin published in April 2019 by the Ministry of Health of Brazil reported that between December 30, 2018 and March 23, 2019, there were 273,193 possible cases of dengue fever in Brazil (estimated incidence of 131 cases/100,000 inhabitants), with 210 confirmed serious cases and 80 deaths^11^. In the same bulletin, the State of Minas Gerais counted 67,363 new cases of dengue fever with an estimated incidence of 320.2 cases/100,000 inhabitants^11^.

Concerning the pathophysiology of SCD, HbS polymerization, vaso-occlusion, and hemolytic anemia are the main factors responsible for both acute and chronic clinical manifestations, and lead to a continuous state of endothelial dysfunction, functional nitric oxide deficiency, persistent oxidative state, sterile systemic inflammation, hypercoagulability, increased neutrophil adhesion, and platelet activation^12^. Due to the estimated prevalence of SCD and the occurrence of regular dengue epidemics in Brazil, dengue infection in patients with SCD is a challenging scenario due to the potentially high risk of systemic complications associated with the sickling process triggered by dengue infection. Patients infected with the dengue virus and SCD patients share endothelial dysfunction as the main mechanism for the occurrence of clinical manifestations. The increase in vascular permeability during dengue infection results from the activation of endothelial cells, and the possible mechanisms involved are direct viral infection or the release of multiple cytokines by infected immune cells^13^.

The interaction of these factors with the complex pathophysiology surrounding dengue virus infection, with special emphasis on endothelial damage/dysfunction and platelet activation, may predispose to the development of organ dysfunction, as seen in the two reported cases. However, the reasons for the lack of increase in the admission of SCD patients with severe and acute hepatobiliary complications (and other severe acute sickle cell crises) triggered by dengue fever are not known. The findings described here have not been previously reported after an extensive literature review.

In conclusion, severe and acute complications of SCD can be triggered by dengue virus infection. It is important to emphasize that the frontline doctors of the emergency units know how to recognize early unusual and potentially fatal complications in SCD patients and complications that can be triggered by dengue infection.
